# Generation of Lung Epithelium from Pluripotent Stem Cells

**DOI:** 10.1007/s40139-013-0016-9

**Published:** 2013-04-03

**Authors:** Amy P. Wong, Janet Rossant

**Affiliations:** 1 Program in Developmental & Stem Cell Biology, Hospital for Sick Children, Toronto, ON M5G 1L7 Canada; 2 Department of Molecular Genetics, University of Toronto, Toronto, ON M5S 1A8 Canada; 3 Hospital for Sick Children, 555 University Avenue, Toronto, ON M5G 1X8 Canada

**Keywords:** Lung, Embryonic stem cells, Induced pluripotent stem cells, Cystic fibrosis, Lung development, Airway epithelium, Pathobiology

## Abstract

The understanding of key processes and signaling mechanisms in lung development has been mainly demonstrated through gain and loss of function studies in mice, while human lung development remains largely unexplored due to inaccessibility. Several recent reports have exploited the identification of key signaling mechanisms that regulate lineage commitment and restriction in mouse lung development, to direct differentiation of both mouse and human pluripotent stem cells towards lung epithelial cells. In this review, we discuss the recent advances in the generation of respiratory epithelia from pluripotent stem cells and the potential of these engineered cells for novel scientific discoveries in lung diseases and future translation into regenerative therapies.

## Introduction

Lung diseases such as cystic fibrosis (CF), chronic obstructive pulmonary disease, and idiopathic pulmonary fibrosis are major health issues for children and adults. Currently the biological understanding of the etiology of many of these diseases is still minimal. Therapeutic interventions are largely based on treating symptoms, not addressing root causes. While such therapies have improved outcomes and increased quality of life, long-term outcomes are still poor. Presently, lung transplantation is the treatment of choice for end-stage lung disease. However, the shortage of donor lungs and the increased risk of secondary complications such as graft rejection and failure [1–3] means that transplantation is a temporary fix.

New strategies to use stem cells to regenerate or generate new lung epithelia have been of growing interest. This review discusses some of the most recent advances in generating lung epithelial cells from pluripotent stem cells and how these newly engineered cells can be used to find new therapies for lung diseases.

### Embryonic Origins of the Lung

**Fig. 1:**
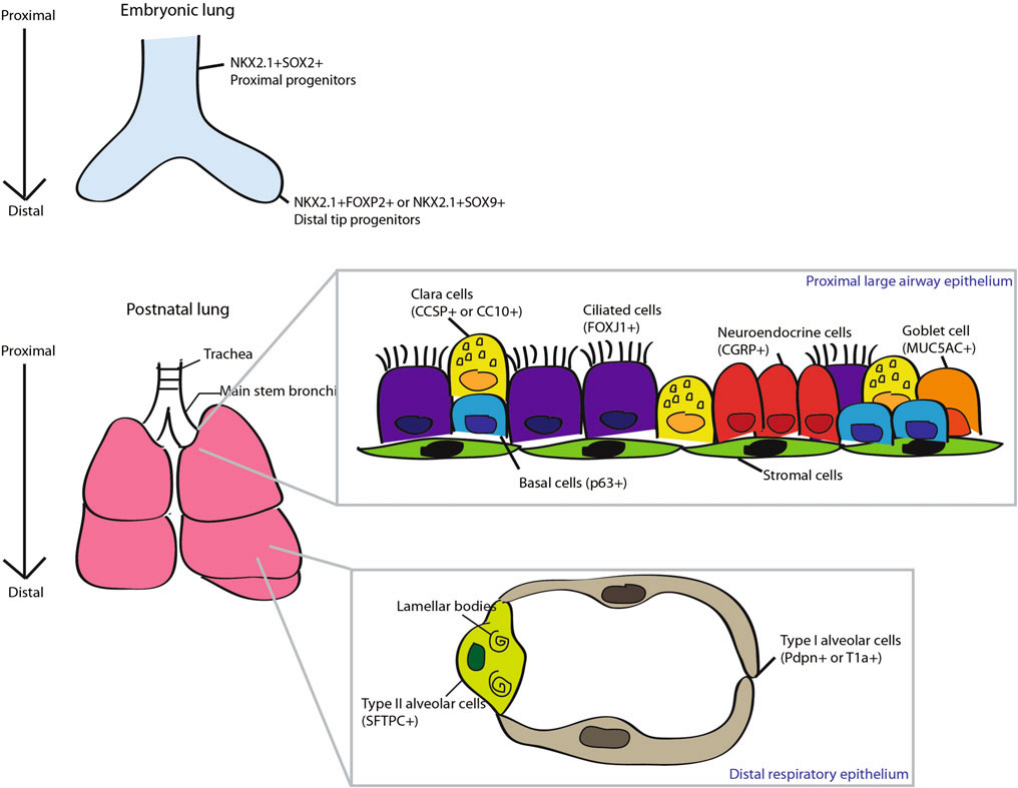
The lung at embryonic and adult stages. In the developing lung (*top panel*), proximal progenitors expressing NKX2.1+ SOX2+ gives rise to proximal cells lineages observed in the adult epithelium (*bottom panel*). The distal tip progenitors marked by NKX2.1+ FOXP2+ or NKX2.1+ SOX9+ contribute to stereotypical branching morphogenesis and eventually the respiratory epithelial cells

The lung is derived from the foregut endoderm. Several patterning events cause separation from the gut tube and other endodermal organs (e.g., liver, pancreas), ultimately leading to the formation of the trachea and lung buds [4, 5•]. Active reciprocal signaling between the developing multipotent distal tip epithelium and surrounding mesenchyme are required for the stereotypical branching morphogenesis of the lung buds, as well as early differentiation events leading to different cell lineages. The proximal epithelium forms first from proximal progenitors with the emergence of neuroendocrine, basal, ciliated, and secretory cells lining the maturing epithelium (Fig. 1). As branching morphogenesis continues, the bronchioles eventually branch into millions of terminal air sacs, or alveoli, where gaseous exchange will take place after birth. Right before birth, the respiratory epithelium is composed mainly of Type I alveolar epithelial cells for gas exchange and Type II cells which secrete the surfactants required to promote breathing at birth. Completion of lung maturation occurs post-natally.

As the lung matures, cell turnover decreases [6] and is very slow in the fully mature adult lung [7]. While studies have shown some regenerative potential of the fetal and adult lung following injury [8–12], failure of the lung to repair itself often results in chronic inflammation, disease progression and pathogenesis. Strategies to repair injured lungs, by activating resident lung stem cells [13–16] or by grafting bone marrow-derived stem cells [17–19] have shown some success but need to be further validated. A more direct approach based on driving differentiation of pluripotent stem cells towards the different lung epithelial cell types clearly holds promise. However, early efforts to differentiate embryonic stem cells into pulmonary cells showed limited success [20–23]. More recent studies have taken a step-wise approach based on mimicking the stages of normal lung development and have achieved more promising results. Here we provide a brief overview of these studies, and discuss the potential use of these cells for regenerative medicine and drug discovery. For more detailed descriptions of early endoderm and embryonic lung development please refer to other reviews [5•, 24•, 26•, 27]

### Comparative Lung Development

While the phases of lung development are similar between mice and human, the timelines of lung development are quite different. In the mouse, embryonic lung development begins around embryonic day 9 and maturation of the lung is complete about 30 days postnatally. In contrast, each phase of fetal lung development in humans is measured in weeks and complete maturation of the airways is not achieved until up to 2 years post-natally [26•]. In addition, there are clear differences in anatomical and regional distribution of the cell types from the large airways through to the intralobar airway epithelium between mouse and human [12, 28–33]. The current knowledge of lung development is largely based on gain- and loss-of-function studies in mice [5•, 25, 26•, 27, 34, 35]. This knowledge has been the basis of attempts at directed differentiation of stem cells to lung epithelium. Comparative studies of lung differentiation from mouse and human pluripotent cells have suggested that the general rules of differentiation are similar. However, the fine details may differ in ways that could have significance for the sought-for regenerative outcomes. Careful study of the development of different lung cell types from stem cells of mouse and human origin may help reveal the underlying similarities and differences in terms of timing, marker expression and potentially even differentiation pathways.

### Directed Differentiation of Pluripotent Stem Cells to Lung Epithelial Cells

**Fig. 2:**
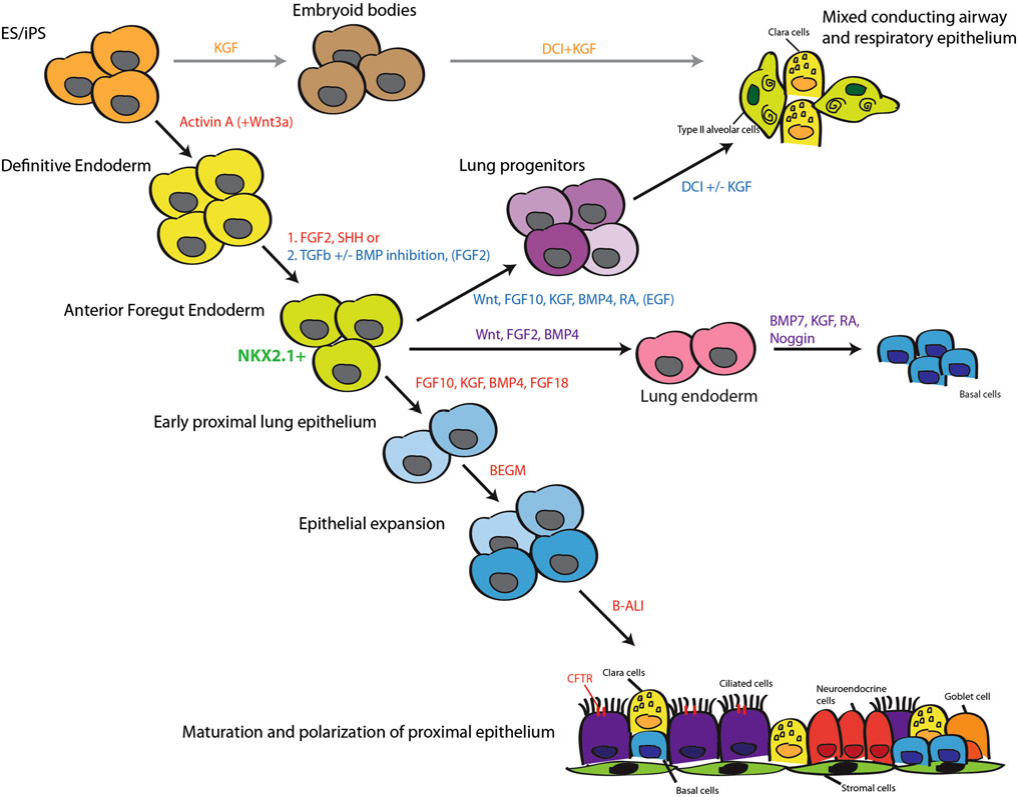
Differentiation strategies to generate pulmonary epithelial cell lineages from pluripotent stem cells. *Arrows* demarcate the lineages achieved from each step. The supplements used for the generation of a particular cell lineage is demarcated by the same color-code. A new color-code for the supplements used means the differentiation step drives the generation of a separate reported cell population but the preceding pathway(s) are the same as indicated

The first indication of the developing embryonic lung endoderm is the expression of the transcription factor Nkx2.1 (or TTF1), in the anterior foregut compartment. The first hurdle, therefore, in generating lung epithelial cells from pluripotent stem cells is to establish an Nkx2.1-expressing embryonic lung endoderm from definitive endoderm (Fig. 2). The first study to show directed differentiation of human embryonic stem cells into anterior foregut endoderm progenitors was published by Green et al. [36••] in early 2011. Using a well established method to generate definitive endoderm [37] with high concentration of Activin A, Green et al. [36••] demonstrated that inhibition of both the BMP and TGF-β signaling pathway using NOGGIN (a physiological inhibitor) and SB431452 (a pharmacological inhibitor), respectively, can induce differentiation of definitive endoderm cells into anterior foregut endoderm (AFE). Up-regulated expression of the foregut marker SOX2 and a concomitant down-regulation of the hindgut marker CDX2 were observed. Dorsoventral patterning of the anterior foregut gives rise to the dorsal esophagus and the ventral lungs and trachea. To specify the lung from the AFE cells, the authors used a combination of Wnt3a, FGF10, KGF, BMP4 and EGF to generate up to 37 % NKX2.1+ lung cells. Addition of retinoic acid induced expression of the classical Type II alveolar cell marker surfactant protein-C (SFTPC) and the ciliated cell marker FOXJ1. This was the first study to present a method to enrich for AFE cells, which can then be directed to differentiate into more mature AFE-derived lineages.

Using embryonic stem cells derived from a mouse line carrying an Nkx2.1–GFP reporter, Longmire et al. [38••] adopted the step-wise protocol from Green et al. [36••] to generate ventral foregut endoderm with the exception that they also included a high concentration of FGF2. Interestingly, exposure of definitive endoderm cells to NOGGIN and SB431452 alone was sufficient to induce GFP expression in up to 21 % of the cells. Gene expression profiling of the sorted GFP+ cells after treatment with Wnt3a, FGF10, KGF, BMP4, EGF and FGF2 revealed up-regulated expression of both lung and thyroid lineage genes. Further differentiation of the cells with FGF2, FGF10, and a mixture of KGF, dexamethasone, cAMP and IBMX (also known as DCI and previously shown to induce transcriptomic changes in fetal lung epithelial cells [39, 40]), resulted in down-regulation of Nkx2.1 in approximately half of the cells. Of the Nkx2.1-negative population, up to 40 % expressed a marker associated with Type I alveolar cells (Pdpn or T1a). Of the Nkx2.1-positive cells, some cells expressed the pro-form of SFTPC, suggestive of Type II cells. Recellularization of decellularized mouse lungs with Nkx2.1–GFP+ cells showed some engraftment of these donor cells as Type I T1a-expressing cells in the parenchyma.

Mou et al. [41••] used a slightly different approach to generate multipotent lung and airway progenitors from mouse and human pluripotent stem cells. Starting with a monolayer method of differentiation, Mou et al. [42]. adapted a previously published method of generating definitive endoderm with high efficiency. Unlike the studies by Green et al., and Longmire et al., TGFβ inhibition with SB431452 alone was sufficient to induce anterior patterning of the definitive endoderm cells. Following the addition of BMP4, FGF2 and a GSK3 inhibitor, up to 10 and 30 % of Nkx2.1 expressing cells were observed with mouse cells and human induced pluripotent stem (iPS) cells, respectively. To generate airway progenitors from the Nkx2.1-expressing cells, a combination of retinoic acid, BMP7, KGF, Wnt antagonism and MAPK/ERK inhibition was used. This produced up to 18 % Nkx2.1+ Sox2+ proximal progenitors in the population of cells. Of the total Nkx2.1+ population, a smaller percentage (1–4 %) also expressed p63, a marker associated with conducting airway basal cells [29]. Interestingly, using an in vivo model of differentiation with a mixed population of lung endoderm cells in matrigel and injected subcutaneously in immunodeficient mice, epithelial spheres were observed that contained Clara cell, ciliated cell, goblet cell and basal cell lineages. The efficiency of differentiation both in vivo and in vitro appears low and while proximal cell lineages were established, distal lung parenchymal epithelia characterized by Type I and Type II cells were not generated even though Nkx2.1+ Sox9+ or Nkx2.1+ FoxP2+ multipotent distal progenitor cells were established.

Two recent publications have bypassed the early stepwise differentiation process and showed some success in generating distal respiratory epithelial cell types. Schmeckebier et al. [43] recently reported that KGF, a known epithelial mitogen [44] that can promote maturation of Type II fetal rat alveolar cells [45], can induce differentiation and maturation of mouse ES and iPS-derived Type II cells in combination with glucocorticoids, cAMP-derivatives and compounds that elevate cAMP levels. While Longmire et al., used DCI and KGF to induce alveolar differentiation from lung endoderm progenitors, Schmeckebier et al., added KGF early in the differentiation process from embryoid bodies, followed by addition of DCI and KGF at day 14. They report a 14-fold greater expression of SP-C and fivefold greater expression of aquaporin-5 (Type I alveolar cell marker) compared to unstimulated controls within 10 days. Electron microscopy revealed features of Type II alveolar cell structures such as apical microvilli and electron-dense lamellar bodies.

Another study by Siti-Ismail et al. [46] reported an impressive ability to generate Type II cells that does not rely on addition of defined exogenous growth factors, but rather on a bioengineering process utilizing encapsulation of mES cells in hydrogels and culture in conditioned media from an alveolar cancer cell line (A549) in a rotary bioreactor [47]. In as little as 5 days, up to 50 % of the cells were reported to be Type II alveolar cells that under electron microscopy exhibited ultrastructural features such as microvilli and lamellar bodies. Furthermore, these cells could be maintained in the bioreactor for 100 days and plated onto 2D cultures without differentiating into Type I cells. It would be interesting to know how well these engineered Type II cells integrate and function in vivo in respiratory epithelia and whether human pluripotent stem cells can also be differentiated as efficiently using this method.

Rather than focus on distal alveolar epithelium, we recently outlined an in vitro differentiation protocol for generating proximal airway epithelia from human pluripotent stem cells, the cell population that is most affected in CF patients. The cystic fibrosis transmembrane conductance regulator (CFTR) protein channel [48••] is involved in chloride and water transport across the airway epithelium. Mutations in this gene lead to failure to maintain proper mucociliary transport and clearance of pathogens. In our step-wise differentiation process, pluripotent stem cells were induced to differentiate progressively into definitive endoderm, anterior foregut endoderm, proximal epithelial progenitors, followed by proliferation, maturation and polarization of the proximal epithelia in an air–liquid interface. The result was maturation of patches of tight junction-coupled differentiated airway epithelial cells. These cells showed up-regulated expression of several characteristic proximal large airway markers, including the polarized apical localization of the CFTR protein needed for proper chloride transport. The CFTR transport functions were active in the differentiated cells as demonstrated by responsiveness to cAMP agonists and a CFTR potentiator in a modified iodide efflux assay for CFTR activity. This study also showed generation of mature airway epithelia from human iPS cells, derived from CF patients. Analysis of these cells shows great promise to study the role of unique genetic modifiers in CFTR activity [49–51] especially since treatment of the CF-iPS-derived airway epithelial cells with a small molecule compound, C18, resulted in partial correction of the CFTR protein in the plasma membrane, suggesting that these cells could be used to model CF in vitro.

It should be noted, however, that this protocol, while successful in generating around 40–50 % CFTR+ polarized lung epithelial cells, still results in a heterogeneous mixture of other endoderm cell types. To date, there has not been a lung differentiation protocol reported that produces a homogeneous cell type of interest. Future refinements, including sorting appropriate cells at appropriate stages of the differentiation protocol, will be needed to solve the heterogeneity issue. Furthermore, while functional activity of CFTR showed levels similar to control epithelial cell lines, future studies will also need to determine the similarities between CFTR function from iPS-derived cells compared to normal post-natal bronchial primary epithelial cells.

### Application of Engineered Lung Cells

#### Disease Modeling

Recent advances in generating airway and distal lung epithelial cells and the ability to generate a renewable source of these cells in vitro offers great hope in using these cells to study lung diseases. Reprogramming of somatic cells into induced pluripotent stem (iPS) cells has created a powerful and unlimited source of patient-specific cells bearing many pulmonary congenital defects [41••, 48••, 52]. These cells carry the mutation that causes or is associated with the disease and can be used to model the disease in vitro. The ability to generate tissue-specific cells from iPS cells offers great opportunities to model many lung diseases in vitro, including the pathogenesis of lung diseases caused by respiratory infections and other congenital defects such as surfactant protein deficiency [53]. For the latter, the lack of reliable methods to generate Type II alveolar cells from human iPS cells remains an impediment to progress towards an in vitro model of this lung disease. A promising example of an application of iPS for disease modeling is CF, which is the most common life shortening congenital disease amongst Caucasians. The disease affects epithelial tissues lining multiple organs throughout the body, including the airways, skin, intestines, pancreatic ducts, and reproductive organs. Lack of ion transport across the airway epithelium leads to improper airway fluid balance, mucous thickening and defective mucociliary clearance, thereby creating a niche for chronic bacterial infections in the airways. These infections are the main cause of mortality in CF patients. Modeling CF has been difficult. The traditional CF knockout mouse model does not recapitulate the pulmonary aspect of the disease, and the availability of patient-specific CF lung epithelium is limiting for in vitro studies. Other animal models of CF, such as swine [54] and ferret [55], display airway phenotypes similar to humans and are useful surrogates for some human studies. However, they cannot model the genetic heterogeneity in disease outcome shown in humans. Patients carrying the same CF mutation will have varying degrees of disease progression and responses to therapy. This is in part due to genetic modifiers that have been shown to affect disease progression [49, 56, 57]. Generation of CF patient iPS cell-derived airway cells in vitro is thus a critical tool for studying the molecular pathways, the effects of environmental insults and the genetic modifiers of CF pathogenesis.

### Drug Screening

Patient-derived induced iPS cells hold great promise for patient-specific drug discovery. The most common CF mutation (~70 % of cases) in the CF gene is caused by a trinucleotide in-frame deletion at position 508 (F508del) of the peptide sequence [58]. Consequently the mutant CFTR protein does not fold properly in the endoplasmic reticulum and is rapidly targeted for degradation instead of translocating to the cell membrane for chloride transport. Recent studies have shown that small molecules called “corrector” compounds are effective in rescuing some of the mutant defects in protein processing and trafficking [59–61]. As a proof-of-concept, we recently reported that CF-iPSC-derived lung epithelial cells could be used to validate novel CF corrector compounds. An active analog of the small molecule VX-809 (vertex 809 currently in phase II clinical trials) promoted plasma membrane accumulation of the mature complex glycosylated form of CFTR protein (band C) in F508del CF-iPS cell-derived lung epithelial cells [48••]. The CF patient iPS cell-derived airway epithelial cells therefore provide a novel platform for cell-based patient-specific screens to find new treatments for CF and other airway diseases [49, 56, 57]

Small molecule screening could also be used to identify novel compounds that improve the directed differentiation of iPS cells to lung epithelial cells. Current differentiation methods use recombinant proteins from xenobiotic sources (bacteria or insect cells) and have high batch to batch variability. In addition, the cost of these recombinant proteins can be astronomical if scale-up of the differentiation process was needed for regenerative purposes. Small molecules, therefore, can be used to improve and control the cell differentiation process. Use of less economical, but highly efficient and reproducible compounds, has been previously shown by Borowiak et al. [62] in driving definitive endoderm in mouse and human ESC and by Chen et al. [63] in driving human ESC into pancreatic progenitors. Small molecules can theoretically be used to generate specific lung progenitor populations or direct certain lung lineages over others.

Overall, the ability to generate a renewable source of airway epithelial cells from pluripotent stem cells holds great promise for high-content screens for therapy.

### Lung Regeneration

Cell replacement strategies have shown some success in several models of lung injury [64–66]. Regeneration by extrapulmonary sources such as the bone marrow [18, 67–69] and cord blood [70, 71] have identified these cells as potential sources of stem cells for therapeutic strategies. Bioengineering of lung organoids using simple biodegradable scaffolds seeded with fetal lung cells [72–74] is also a promising new avenue for tissue replacement approaches. However, use of these scaffolds to differentiate and support non-lung-derived cells such as pluripotent stem cells has yet to be determined. One of the limitations in these organoid cultures is the importance of mechanical stretch in activating pathways involved in epithelial differentiation and proliferation [75–77].

Recent advances in the decellularization of the lung may provide a more plausible system since most of the lung extracellular matrices are preserved and allow initial binding of the donor cells to the lung tissue [78–81]. In 2010, two seminal papers by Ott et al. [82] and Petersen et al. [83] demonstrated that cadaveric rat lungs could be safely decellularized leaving most of the extracellular matrix intact and then using a bioreactor, recellularized with epithelial and endothelial cells. Petersen et al. [83] showed that the engineered lungs had regional-specific reorganization of the cells mechanical properties similar to native lung tissue. Furthermore, both papers demonstrated that when these engineered lungs were orthotopically transplanted short-term into a recipient rat, the lungs participated in gas exchange. These two publications has since spurred many publications demonstrating reconstitution of the decellularized lung with a variety of cell types, including mesenchymal stem cells [79, 80] and fetal lung cells [78]. The use of pluripotent stem cell-derived cells in recellularizing the lungs is promising since these cells offer an unlimited supply of autologous cells for repopulation.

In the excitement around the decellularized lung model, it is worth remembering that the adult lung is comprised of at least 40 different cell types with specialized functions including gas exchange, metabolism of xenobiotics, and immunity to name a few. Therefore, to completely recapitulate the cell lineages and functions of the lung in a decellularized model and then use these lungs as replacement organs in transplantation remains a distant horizon. However, as more studies identify methods to generate various mature lung cell types or early lung progenitor cells that can potentially differentiate into several cell lineages in vivo (Fig. 2), perhaps partial regeneration of the lung can be accomplished.

The recent success of a main stem bronchus replacement in 2008 with a decellularized human donor trachea recellularized with the recipient’s own respiratory epithelial and stem cell-derived chondrocytes [84] has caused much excitement in engineering bioartificial airways as replacement strategies for lung diseases. The same group recently demonstrated the clinical success of transplanting a “bioartificial nanocomposite” seeded with the patient’s bone marrow mononuclear cells that partly regenerated the tracheobronchial airway with healthy epithelium after 5 months [85]. Long-term follow-up is yet unknown in these patients and the mechanism of how these cells regenerate remains unclear. A fuller understanding of this process might provide clues as to how to promote endogenous repopulation of other more complex organs, based around decellularized or artificial matrices.

### Potential of Direct Conversion of Fibroblasts to Epithelial Cells

There is some evidence for direct conversion of fibroblasts into neurons [86], cardiomyocytes [87] and hematopoietic cells [88], based on the introduction of the four reprogramming transcription factors (OCT4, CMYC, SOX2, KLF4) combined with microenvironmental cues to direct lineage commitment. It is believed that the reprogramming regimen resets the epigenetic fate map of the cells rendering the cells in an epigenetically unstable state. This then allows the influence of extracellular cues such as cell culture conditions to select for or direct phenotypic changes of these cells in their new microenvironment. It has yet to be shown that this direct conversion or “transdifferentiation” of cells can be done using conditions that would support airway or lung epithelial cell growth. This avenue of research is certainly worth exploring as it could avoid the long and complex process of maturing lung cell types from embryonic precursors.

## Conclusion

Current methods of generating lung epithelial cells from pluripotent stem cells are not 100 % efficient and cultures are often contaminated with other endodermal cell types, making it difficult to use these cells for reliable high-content drug screens and tissue regeneration. Future efforts will need to identify methods to isolate embryonic lung progenitor cells from the differentiating cultures or identify methods to generate postnatal stem cells that can differentiate into all lung cell types in vitro. In addition, in vivo assessment of the potential to functionally integrate with native cells and generate mature lung epithelial cell lineages should become the gold standard for validating any lung cell populations generated in vitro.

## References

[1] Porhownik NR (2013). Airway complications post lung transplantation. Curr Opin Pulm Med.

[2] Sato M, Hwang DM, Waddell TK, Singer LG, Keshavjee S (2013). Progression pattern of restrictive allograft syndrome after lung transplantation. J Heart Lung Transplant.

[3] Sato M, Hwang DM, Ohmori-Matsuda K (2012). Revisiting the pathologic finding of diffuse alveolar damage after lung transplantation. J Heart Lung Transplant.

[4] Que J, Choi M, Ziel JW, Klingensmith J, Hogan BL (2006). Morphogenesis of the trachea and esophagus: current players and new roles for noggin and Bmps. Differentiation.

[5] • Morrisey EE, Hogan BL (2010) Preparing for the first breath: genetic and cellular mechanisms in lung development. Dev Cell 18:8–23. *A very detailed review of lung development*10.1016/j.devcel.2009.12.010 PMC3736813 20152174

[6] Kauffman SL (1975). Kinetics of pulmonary epithelial proliferation during prenatal growth of the mouse lung. Anat Rec.

[7] Kauffman SL (1980). Cell proliferation in the mammalian lung. Int Rev Exp Pathol.

[8] Kauffman SL (1977). Proliferation, growth, and differentiation of pulmonary epithelium in fetal mouse lung exposed transplacentally to dexamethasone. Lab Invest.

[9] Evans MJ, Cabral LJ, Stephens RJ, Freeman G (1973). Renewal of alveolar epithelium in the rat following exposure to NO_2_. Am J Pathol.

[10] Evans MJ, Shami SG, Cabral-Anderson LJ, Dekker NP (1986). Role of nonciliated cells in renewal of the bronchial epithelium of rats exposed to NO_2_. Am J Pathol.

[11] Peake JL, Reynolds SD, Stripp BR, Stephens KE, Pinkerton KE (2000). Alteration of pulmonary neuroendocrine cells during epithelial repair of naphthalene-induced airway injury. Am J Pathol.

[12] Van Winkle LS, Buckpitt AR, Nishio SJ, Isaac JM, Plopper CG (1995). Cellular response in naphthalene-induced Clara cell injury and bronchiolar epithelial repair in mice. Am J Physiol.

[13] McQualter JL, Yuen K, Williams B, Bertoncello I (2010). Evidence of an epithelial stem/progenitor cell hierarchy in the adult mouse lung. Proc Natl Acad Sci USA.

[14] Teisanu RM, Lagasse E, Whitesides JF, Stripp BR (2009). Prospective isolation of bronchiolar stem cells based upon immunophenotypic and autofluorescence characteristics. Stem Cells.

[15] Kim CF, Jackson EL, Woolfenden AE (2005). Identification of bronchioalveolar stem cells in normal lung and lung cancer. Cell.

[16] Giangreco A, Arwert EN, Rosewell IR, Snyder J, Watt FM, Stripp BR (2009). Stem cells are dispensable for lung homeostasis but restore airways after injury. Proc Natl Acad Sci USA.

[17] Wong AP, Keating A, Lu WY (2009). Identification of a bone marrow-derived epithelial-like population capable of repopulating injured mouse airway epithelium. J Clin Invest.

[18] Gomperts BN, Belperio JA, Rao PN (2006). Circulating progenitor epithelial cells traffic via CXCR4/CXCL12 in response to airway injury. J Immunol.

[19] Macpherson H, Keir P, Webb S (2005). Bone marrow-derived SP cells can contribute to the respiratory tract of mice in vivo. J Cell Sci.

[20] Rippon HJ, Ali NN, Polak JM, Bishop AE (2004). Initial observations on the effect of medium composition on the differentiation of murine embryonic stem cells to alveolar type II cells. Cloning Stem Cells.

[21] Van Vranken BE, Rippon HJ, Samadikuchaksaraei A, Trounson AO, Bishop AE (2007) The differentiation of distal lung epithelium from embryonic stem cells, Chap. 1, Unit 1G. Curr Protoc Stem Cell Biol. doi:10.1002/9780470151808.sc01g01s2 10.1002/9780470151808.sc01g01s2 18785171

[22] Samadikuchaksaraei A, Cohen S, Isaac K (2006). Derivation of distal airway epithelium from human embryonic stem cells. Tissue Eng.

[23] Ali NN, Edgar AJ, Samadikuchaksaraei A (2002). Derivation of type II alveolar epithelial cells from murine embryonic stem cells. Tissue Eng.

[24] • Zorn AM, Wells JM (2009) Vertebrate endoderm development and organ formation. Annu Rev Cell Dev Biol 25:221–251. *A very detailed review of early endoderm development and the lineages that are derived from it*10.1146/annurev.cellbio.042308.113344 PMC2861293 19575677

[25] Cardoso WV (2001). Molecular regulation of lung development. Annu Rev Physiol.

[26] • Kimura J, Deutsch GH (2007) Key mechanisms of early lung development. Pediatr Dev Pathol 10:335–347. *A very nice review of the key signalling pathways involved in lung development* 10.2350/07-06-0290.1 17929994

[27] Kumar VH, Lakshminrusimha S, El Abiad MT, Chess PR, Ryan RM (2005). Growth factors in lung development. Adv Clin Chem.

[28] Ochs M, Nyengaard JR, Jung A (2004). The number of alveoli in the human lung. Am J Respir Crit Care Med.

[29] Rock JR, Onaitis MW, Rawlins EL (2009). Basal cells as stem cells of the mouse trachea and human airway epithelium. Proc Natl Acad Sci USA.

[30] Soutiere SE, Tankersley CG, Mitzner W (2004). Differences in alveolar size in inbred mouse strains. Respir Physiol Neurobiol.

[31] Boers JE, Ambergen AW, Thunnissen FB (1998). Number and proliferation of basal and parabasal cells in normal human airway epithelium. Am J Respir Crit Care Med.

[32] Boers JE, Ambergen AW, Thunnissen FB (1999). Number and proliferation of Clara cells in normal human airway epithelium. Am J Respir Crit Care Med.

[33] Nakajima M, Kawanami O, Jin E (1998). Immunohistochemical and ultrastructural studies of basal cells, Clara cells and bronchiolar cuboidal cells in normal human airways. Pathol Int.

[34] Whitsett JA, Haitchi HM, Maeda Y (2011). Intersections between pulmonary development and disease. Am J Respir Crit Care Med.

[35] Cardoso WV, Lu J (2006). Regulation of early lung morphogenesis: questions, facts and controversies. Development.

[36] •• Green MD, Chen A, Nostro MC, et al (2011) Generation of anterior foregut endoderm from human embryonic and induced pluripotent stem cells. Nat Biotechnol 29:267–272. *This was the first study to demonstrate the ability to make anterior ventral foregut endoderm from human pluripotent stem cells using a directed differentiation approach that mimic*ked in vivo *developmental pathways*10.1038/nbt.1788 PMC4866999 21358635

[37] Gadue P, Huber TL, Paddison PJ, Keller GM (2006). Wnt and TGF-beta signaling are required for the induction of an in vitro model of primitive streak formation using embryonic stem cells. Proc Natl Acad Sci USA.

[38] •• Longmire TA, Ikonomou L, Hawkins F, et al (2012) Efficient derivation of purified lung and thyroid progenitors from embryonic stem cells. Cell Stem Cell 10:398–411. *Using an in*-*house generated mouse reporter Nkx2.1*–*gfp to establish a differentiation method from ES cells, this report illustrates a protocol to generate both lung and thyroid progenitor cells that can be further differentiated into mixed conducting airway and alveolar epithelial cells. With these reporter lines, this group can now perform arrays to identify new cell surface markers to isolate progenitors at various stages of the differentiation*10.1016/j.stem.2012.01.019 PMC3322392 22482505

[39] Gonzales LW, Guttentag SH, Wade KC, Postle AD, Ballard PL (2002). Differentiation of human pulmonary type II cells in vitro by glucocorticoid plus cAMP. Am J Physiol Lung Cell Mol Physiol.

[40] Whitsett JA, Pilot T, Clark JC, Weaver TE (1987). Induction of surfactant protein in fetal lung. Effects of cAMP and dexamethasone on SAP-35 RNA and synthesis. J Biol Chem.

[41] •• Mou H, Zhao R, Sherwood R, et al (2012) Generation of multipotent lung and airway progenitors from mouse ESCs and patient-specific cystic fibrosis iPSCs. Cell Stem Cell 10:385–397. *A method to generate distal and proximal airway epithelial cell progenitors is outlined. A method to isolate, purify and expand these progenitor cells have yet to be shown but this study illustrates a method to generate both mouse and human progenitors with greater efficiencies using a modified approach of Green* et al. *and Longmire* et al. 10.1016/j.stem.2012.01.018 PMC3474327 22482504

[42] Sherwood RI, Maehr R, Mazzoni EO, Melton DA (2011). Wnt signaling specifies and patterns intestinal endoderm. Mech Dev.

[43] Schmeckebier S, Mauritz C, Katsirntaki K (2013). Keratinocyte growth factor and dexamethasone plus elevated cAMP levels synergistically support pluripotent stem cell differentiation into alveolar epithelial type II cells. Tissue Eng A.

[44] Shiratori M, Oshika E, Ung LP (1996). Keratinocyte growth factor and embryonic rat lung morphogenesis. Am J Respir Cell Mol Biol.

[45] Chelly N, Mouhieddine-Gueddiche OB, Barlier-Mur AM, Chailley-Heu B, Bourbon JR (1999). Keratinocyte growth factor enhances maturation of fetal rat lung type II cells. Am J Respir Cell Mol Biol.

[46] Siti-Ismail N, Bishop AE, Polak JM, Mantalaris A (2008). The benefit of human embryonic stem cell encapsulation for prolonged feeder-free maintenance. Biomaterials.

[47] Siti-Ismail N, Samadikuchaksaraei A, Bishop AE, Polak JM, Mantalaris A (2012). Development of a novel three-dimensional, automatable and integrated bioprocess for the differentiation of embryonic stem cells into pulmonary alveolar cells in a rotating vessel bioreactor system. Tissue Eng C Methods.

[48] •• Wong AP, Bear CE, Chin S, et al (2012) Directed differentiation of human pluripotent stem cells into mature airway epithelia expressing functional CFTR protein. Nat Biotechnol 30(9):876–882. *A directed stepwise differentiation protocol is outlined to generate proximal airway epithelial cells that express a functional CFTR channel. The study is the first study to show as proof*-*of*-*concept the use of human iPS cell*-*derived lung cells for* in vitro *modeling of CF and drug screening*10.1038/nbt.2328 PMC3994104 22922672

[49] Wright FA, Strug LJ, Doshi VK (2011). Genome-wide association and linkage identify modifier loci of lung disease severity in cystic fibrosis at 11p13 and 20q13.2. Nat Genet.

[50] Gisler FM, von Kanel T, Kraemer R, Schaller A, Gallati S (2012). Identification of SNPs in the cystic fibrosis interactome influencing pulmonary progression in cystic fibrosis. Eur J Hum Genet.

[51] Corvol H, Boelle PY, Brouard J (2008). Genetic variations in inflammatory mediators influence lung disease progression in cystic fibrosis. Pediatr Pulmonol.

[52] Somers A, Jean JC, Sommer CA (2010). Generation of transgene-free lung disease-specific human induced pluripotent stem cells using a single excisable lentiviral stem cell cassette. Stem Cells.

[53] Wert SE, Whitsett JA, Nogee LM (2009). Genetic disorders of surfactant dysfunction. Pediatr Dev Pathol.

[54] Pezzulo AA, Tang XX, Hoegger MJ (2012). Reduced airway surface pH impairs bacterial killing in the porcine cystic fibrosis lung. Nature.

[55] Sun X, Sui H, Fisher JT (2010). Disease phenotype of a ferret CFTR-knockout model of cystic fibrosis. J Clin Invest.

[56] Sun L, Rommens JM, Corvol H (2012). Multiple apical plasma membrane constituents are associated with susceptibility to meconium ileus in individuals with cystic fibrosis. Nat Genet.

[57] Taylor C, Commander CW, Collaco JM (2011). A novel lung disease phenotype adjusted for mortality attrition for cystic fibrosis genetic modifier studies. Pediatr Pulmonol.

[58] Riordan JR, Rommens JM, Kerem B (1989). Identification of the cystic fibrosis gene: cloning and characterization of complementary DNA. Science.

[59] Wilschanski M (2010). Small molecules to treat cystic fibrosis. Proc Am Thorac Soc.

[60] Corbyn Z (2012). Promising new era dawns for cystic fibrosis treatment. Lancet.

[61] Clancy JP, Rowe SM, Accurso FJ (2012). Results of a phase IIa study of VX-809, an investigational CFTR corrector compound, in subjects with cystic fibrosis homozygous for the F508del-CFTR mutation. Thorax.

[62] Borowiak M, Maehr R, Chen S (2009). Small molecules efficiently direct endodermal differentiation of mouse and human embryonic stem cells. Cell Stem Cell.

[63] Chen S, Borowiak M, Fox JL (2009). A small molecule that directs differentiation of human ESCs into the pancreatic lineage. Nat Chem Biol.

[64] Wong AP, Dutly AE, Sacher A (2007). Targeted cell replacement with bone marrow cells for airway epithelial regeneration. Am J Physiol Lung Cell Mol Physiol.

[65] Seguin A, Baccari S, Holder-Espinasse M (2012). Tracheal regeneration: evidence of bone marrow mesenchymal stem cell involvement. J Thorac Cardiovasc Surg.

[66] Duchesneau P, Wong AP, Waddell TK (2010). Optimization of targeted cell replacement therapy: a new approach for lung disease. Mol Ther.

[67] Chamoto K, Gibney BC, Lee GS (2012). CD34+ progenitor to endothelial cell transition in post-pneumonectomy angiogenesis. Am J Respir Cell Mol Biol.

[68] Kleeberger W, Versmold A, Rothamel T (2003). Increased chimerism of bronchial and alveolar epithelium in human lung allografts undergoing chronic injury. Am J Pathol.

[69] Albera C, Polak JM, Janes S (2005). Repopulation of human pulmonary epithelium by bone marrow cells: a potential means to promote repair. Tissue Eng.

[70] Sueblinvong V, Loi R, Eisenhauer PL (2008). Derivation of lung epithelium from human cord blood-derived mesenchymal stem cells. Am J Respir Crit Care Med.

[71] Montemurro T, Andriolo G, Montelatici E (2011). Differentiation and migration properties of human foetal umbilical cord perivascular cells: potential for lung repair. J Cell Mol Med.

[72] Andrade CF, Wong AP, Waddell TK, Keshavjee S, Liu M (2007). Cell-based tissue engineering for lung regeneration. Am J Physiol Lung Cell Mol Physiol.

[73] Douglas WH, Teel RW (1976). An organotypic in vitro model system for studying pulmonary surfactant production by type II alveolar pneumonocytes. Am Rev Respir Dis.

[74] Simpson LL, Tanswell AK, Joneja MG (1985). Epithelial cell differentiation in organotypic cultures of fetal rat lung. Am J Anat.

[75] Liu M, Xu J, Tanswell AK, Post M (1993). Stretch-induced growth-promoting activities stimulate fetal rat lung epithelial cell proliferation. Exp Lung Res.

[76] Nakamura T, Liu M, Mourgeon E, Slutsky A, Post M (2000). Mechanical strain and dexamethasone selectively increase surfactant protein C and tropoelastin gene expression. Am J Physiol Lung Cell Mol Physiol.

[77] Liu M, Xu J, Souza P, Tanswell B, Tanswell AK, Post M (1995). The effect of mechanical strain on fetal rat lung cell proliferation: comparison of two- and three-dimensional culture systems. In Vitro Cell Dev Biol Anim.

[78] Price AP, England KA, Matson AM, Blazar BR, Panoskaltsis-Mortari A (2010). Development of a decellularized lung bioreactor system for bioengineering the lung: the matrix reloaded. Tissue Eng A.

[79] Bonvillain RW, Danchuk S, Sullivan DE (2012). A nonhuman primate model of lung regeneration: detergent-mediated decellularization and initial in vitro recellularization with mesenchymal stem cells. Tissue Eng A.

[80] Daly AB, Wallis JM, Borg ZD (2012). Initial binding and recellularization of decellularized mouse lung scaffolds with bone marrow-derived mesenchymal stromal cells. Tissue Eng A.

[81] Wallis JM, Borg ZD, Daly AB (2012). Comparative assessment of detergent-based protocols for mouse lung de-cellularization and re-cellularization. Tissue Eng C Methods.

[82] Ott HC, Clippinger B, Conrad C (2010). Regeneration and orthotopic transplantation of a bioartificial lung. Nat Med.

[83] Petersen TH, Calle EA, Zhao L (2010). Tissue-engineered lungs for in vivo implantation. Science.

[84] Macchiarini P, Jungebluth P, Go T (2008). Clinical transplantation of a tissue-engineered airway. Lancet.

[85] Jungebluth P, Alici E, Baiguera S (2011). Tracheobronchial transplantation with a stem-cell-seeded bioartificial nanocomposite: a proof-of-concept study. Lancet.

[86] Kim J, Efe JA, Zhu S (2011). Direct reprogramming of mouse fibroblasts to neural progenitors. Proc Natl Acad Sci USA.

[87] Efe JA, Hilcove S, Kim J (2011). Conversion of mouse fibroblasts into cardiomyocytes using a direct reprogramming strategy. Nat Cell Biol.

[88] Szabo E, Rampalli S, Risueno RM (2010). Direct conversion of human fibroblasts to multilineage blood progenitors. Nature.

